# Lessons drawn from research utilization in the maternal iodine supplementation policy development in Thailand

**DOI:** 10.1186/1471-2458-12-391

**Published:** 2012-05-30

**Authors:** Utsana Tonmukayakul, Román Pérez Velasco, Sripen Tantivess, Yot Teerawattananon

**Affiliations:** 1 Health Intervention and Technology Assessment Program (HITAP), 6th floor, 6th Building, Department of Health, Ministry of Public Health, Tiwanon Road, Muang, Nonthaburi, 11000, Thailand

**Keywords:** Iodine deficiency disorders, Dietary supplementation, Pregnancy, Policy development, Health policy, Research utilization, Case study, Rapid review, Thailand

## Abstract

In this paper, the authors share their experience on evidence utilization in the development of Thailand’s maternal iodine supplementation policy in 2009–2010. Observations and reflections on their experience of engaging with research for policymaking are illustrated. The case study indicates that rapid approaches in conducting research, namely a targeted literature review and cross-sectional survey of professionals’ opinions and current practices were efficient in achieving the timeliness of evidence provision. In addition pro-activity, trust and interaction between researchers and policymakers enhanced the research–policy integration. The Thai experience may be useful for other developing countries which pursue evidence-informed policymaking, despite differences in the health system context.

## Background

### Research utilization in policymaking

Recently, there has been a greater attention to evidence-informed policymaking and the transparency, accountability and inclusiveness of its processes [1]. Although prioritization of research topics and systematic reviews/randomized control trials are considered the best approaches to select and generate evidence, sometimes more rapid approaches to gather information are needed due to cost considerations, limited human resources, and urgent demands [2].

Systematic reviews and primary studies of health research utilization have revealed that there are a number of factors influencing research utilization. These include: (a) interaction and trust between decision-makers and researchers; (b) the quality, timeliness and relevance of the evidence produced; (c) the political environment; (d) the attitude and proficiency of decision-makers; (e) established policy networks; (f) the concurrence of research findings with existing policies, and (g) the social and economic environment [3-6].

### Iodine deficiency disorders in Thailand

Iodine deficiency disorders (IDD) remain an important health problem in Thailand, even though several initiatives have been carried out by the Ministry of Public Health (MoPH) in collaboration with international and domestic organizations for decades. Since the establishment of the National IDD Control Board in 1994, three major measures—Universal Salt Iodization (USI), iodized oil capsules and water iodization—have been introduced. Despite these initiatives, mild to moderate IDD and its consequences still exist [7]. An external review conducted by the International Council for Control of Iodine Deficiency Disorders (ICCIDD) in 2009 indicated the ineffectiveness of the USI and suboptimal iodine nutrition in 60–70% of pregnant Thai women [8]. The dissemination of the review results, in particular the information on the declining intellectual quotient in Thai children, drew the attention of concerned parties including the MoPH, not only to the problem of children’s intelligence but also to its attributable factors and potential solutions. Providing iodine supplements to childbearing and lactating women was identified as a key measure towards reversing this trend.

## Objectives

This paper aims to explore the development of maternal iodine supplementation policy in Thailand between November 2009 and October 2010. It sheds light on the role of scientific evidence and the factors encouraging the utilization of such evidence in the policy decisions. The Thai lessons may be helpful for other developing countries pursuing research–policy nexus in their own health system context.

## Researchers’ role in policy process and evidence generation

1. To understand the policy process and identify the factors influencing evidence uptake, observation of stakeholder consultations and a review of government documents, internal circulars, and e-mail communication were employed.

2. To address particular policy questions, the authors conducted a rapid review on the evidence on appropriate dosage and forms of iodine, and a rapid survey of obstetricians’ current practices, perceptions and preferred formulation of micronutrient supplements.

## Observations and reflections on the experience of research utilization

### Timeline of key events

**Figure 1:**
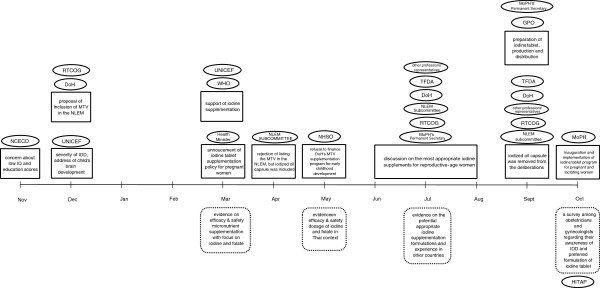
Timeline of key policy development events (November 2009 – October 2010).

The development of the maternal iodine supplementation policy occurred within one year after the findings of the ICCIDD’s review were publicised, as depicted in Figure 1. Key actors are presented in ovals, important events in boxes, and evidence provided in rounded rectangles.

The attention of the Thai government was seriously stimulated in November 2009 when this problematic issue was raised on the meeting agenda of the National Committee on Early Childhood Development (NCECD) [9]. The Committee requested that the MoPH introduce an iodine supplementation program for pregnant women. While this policy was generally agreed upon in principle by most stakeholders, there were questions over the appropriate dosage and formulation of the iodine supplements. In this respect, the Department of Health (DoH) and the Royal Thai College of Obstetricians and Gynecologists (RTCOG) suggested the provision of a multinutrient preparation.

It was not until March 2010 that the MoPH officially announced that it would offer iodine, iron and folic acid tablets to pregnant women, as well as iodine and iron supplements for neonates and children under 5 years old [10]. This initiative received support from the World Health Organization (WHO) and the United Nations Children’s Fund’s (UNICEF) officers in Thailand as the iodine supplement would ensure adequate iodine intake by pregnant and lactating women. Local representatives of the WHO and UNICEF also strongly recommended that the MoPH strengthen the USI program [11,12].

### Devising a policy instrument

However, the new initiative could not get started. Since iodine supplements were not included in the country’s pharmaceutical benefit package, the National List of Essential Medicines (NLEM) [13], the products could not be subsidised in public health schemes. In response to the obstacle, in April 2010 the NLEM Development Committee decided to list iodized oil capsules for maternal use, rather than the multinutrient preparations proposed by the DoH. The three underlying reasons for the decision were: i) a concern for excessive intake of vitamin A during pregnancy (WHO safety limits 10,000 IU), ii) the possibility of overconsumption of unnecessary micronutrients, and iii) the fact that iodized oil capsules were listed in the WHO’s Model List of Essential Medicines. Whereas the DoH and RTCOG argued that a multinutrient preparation would satisfy the nutritional requirements in pregnancy and treatment adherence, both the NLEM and the Universal Coverage Committees rejected this proposal due to the inconclusive evidence.

The debate continued in three consultations chaired by the MoPH’s Permanent Secretary between June and August 2010. The discussions focused on the most appropriate iodine preparations to be offered to pregnant and lactating women. Throughout the consultation processes, several types of evidence were provided by respective organizations. The DoH prepared a list of multinutrient products available in the country, along with a concept note highlighting the crucial roles of folic acid, iodine and iron in child development, as well as the prevalence of iron deficiency anemia and thalassemic pregnancy cases (thalassemia remains an important health problem [14]).

With regards to iodized oil, this formulation was removed from the NLEM in September 2010 because of health professionals’ concerns about potential hyperthyroidism as the preparation was going to be prescribed in a mild-to-moderate iodine deficient setting like Thailand [8].

### The quest for an appropriate preparation

Shortly after, it was concluded that the maternal supplement would only contain folic acid, iodine and iron. The discussion was then geared towards the question of which formulations and dosages should be offered. The options ranged from a combination of the three micronutrients to single tablets of each. At this point, researchers from the Health Intervention and Technology Assessment Program (HITAP), a semi-autonomous research agency under the aegis of the MoPH [15], actively provided evidence synthesized from systematic reviews, clinical trials, guidelines and documents about dosage and formulations for maternal iodine supplementation. Reference lists were scanned, authors of ongoing clinical trials were contacted, and relevant experts were consulted (see Additional file 1).

HITAP had been requested to verify the appropriateness of iodine supplementation, even though this topic was not selected through the conventional prioritization channels. This is because of the trust and interactive relationship built with key policymakers since the agency’s inception [15,16]. Owing to this relationship, HITAP researchers were able to actively share evidence to address questions arising during the deliberations. Also, HITAP proposed to conduct a rapid preference survey among the 2,228 RCOGT members in September 2010 [17] (see Additional file 2).

Drawn from the abovementioned evidence, HITAP recommended a fixed-dose combination of the three nutrients for the target population, and potassium iodide in a single tablet for thalassemic pregnant women. Suggested doses were based on the daily requirements recommended by the WHO and UNICEF ― 400 μg of folic acid, 250 μg of iodine, and 60 μg of iron [18]. Given the current movement to strengthen the USI program, however, some experts suggested that 150 μg was safer than 250 μg of iodine. The Government Pharmaceutical Organization (GPO), a state enterprise under the MoPH [19], was assigned to produce and distribute both preparations. On 1 October 2010, the maternal iodine supplementation program was inaugurated nationwide.

## Discussion and lessons drawn

As suggested in this case study, the decision on the provision of the iodine supplement was made from inception, while evidence from a rapid review and survey played a crucial role in addressing the need for timely evidence on the most suitable iodine-containing preparation.

Rapid reviews and syntheses of selected pieces of information are commonly conducted in response to pressure from decision-makers who frequently need to make decisions in short time periods [2]. Although questions have arisen regarding the appropriateness and transparency of these methodologies in comparison to full reviews, it has been argued that they can still play an important role in evidence-informed decision-making, and emphasis should be placed on their appropriate use [20]. Evidence has shown that the results of rapid and full reviews are not much different, although the scope of the former is narrower [21]. Since the evidential needs of the iodine case deliberations were specifically related to appropriate formulation and dosage, a rapid review appears to be a suitable approach to support policymaking in this instance.

Given the short time available for conducting the survey, the exclusion of non-RTCOG practitioners such as nurses, midwives, and family physicians, all of whom have also provided antenatal care, may imply that maternal iodine supplement prescribers are not perfectly represented. However, specialist physicians have a high influence on other health professionals and patients in Thailand [22,23]; therefore, the survey targeted this group in order to engage them in the future policy implementation.

In addition, the right timing and timeliness of providing research results is critical to foster research utilization as argued in Lavis et al. [4]. In the same vein, this case study suggests that HITAP researchers were able to provide relevant evidence to policymakers and key stakeholders in a timely fashion. This paper also reveals that the pro-active identification of evidence requirements during ongoing policy processes might be a key factor facilitating evidence uptake and strengthening trust and interactive relationships between policymakers and researchers.

## Abbreviations

DoH: Department of Health; GPO: Government Pharmaceutical Organization; HITAP: Health Intervention and Technology Assessment Program; ICCIDD: International Council for Control of Iodine Deficiency Disorders; IDD: Iodine deficiency disorders; MoPH: Ministry of Public Health; NCECD: National Committee on Early Childhood Development; NLEM: National List of Essential Medicines; RTCOG: Royal Thai College of Obstetricians and Gynecologists; TFDA: Thai Food and Drug Administration; UNICEF: United Nations Children’s Fund; USI: Universal Salt Iodization; WHO: World Health Organization.

## Competing interests

The authors declare no conflict of interest.

## Authors’ contributions

All authors contributed equally to the production of this paper.

## Funding

The Health Intervention and Technology Assessment Program (HITAP) is funded by the Thai Health Promotion Foundation; the Health System Research Institute (HSRI); the National Health Security Office (NHSO); and the Bureau of Health Policy and Strategy, Ministry of Public Health. This study did not receive any specific funding. The findings, interpretations, and conclusions expressed in this paper do not necessarily reflect the views of the funding agencies.

## Pre-publication history

The pre-publication history for this paper can be accessed here:

http://www.biomedcentral.com/1471-2458/12/391/prepub

## Supplementary Material

Additional file 1 Summary of the rapid review results Click here for file

Additional file 2 Summary of the rapid review results Click here for file
