# Supplementary material for: Lessons drawn from research utilization in the maternal iodine supplementation policy development in Thailand
Source: BMC Public Health. 2012 May 30;12:391. doi: 10.1186/1471-2458-12-391 (PMC3490728; doi:10.1186/1471-2458-12-391)
Supplement: Additional file 1 — Summary of the rapid review results [file 1471-2458-12-391-S1.doc]

**Additional file 1. Summary of the rapid review results**

**Box 1: Summary of the review findings**

*i) Effectiveness and safety*: Although evidence suggested that maternal supplementation was safe and beneficial, only surrogate markers of cognitive child development were measured. Moreover, evidence on optimal iodine doses and duration of treatment was inconclusive.

*ii) International recommendations*: A number of international organizations have recommended maternal iodine supplementation in mild-to-moderate iodine-deficient settings. At present, only Spain, Macedonia and New Zealand subsidize a maternal supplement. In Spain, a 200 μg iodine-only and a multinutrient tablet are available. New Zealand recommends an iodine-only (150 μg) and a folic acid tablet instead.

*iii) Cost-effectiveness:* There is a wide consensus on fortification (salt iodization, in particular) as the most cost-effective intervention; however, the New Zealander government’s funding decision was supported by a study that showed that, although mandatory fortification was the most cost-effective intervention, adequate maternal urine iodine concentration (UIC) would only be achieved by additional supplementation.

*iv) Appropriateness of dosage forms/formulations:* Iodized oil has been used in moderate-to-severe iodine-deficient regions, remote areas or those without organized healthcare systems. Although its effectiveness and safety has been proven, and cost and treatment compliance are favorable in comparison with other dosage forms, tablets of potassium iodide alone or along with other micronutrients of proven efficacy and safety represent more reliable, higher quality pharmaceutical forms, especially in terms of pharmacokinetics.

**Table 1. Summary of interventions and results from the selected controlled trials**

| **Study (country)** | **Study design** | **Population** | **Intervention (I µg/day )** | **Outcomes** | | **Conclusions** |
| --- | --- | --- | --- | --- | --- | --- |
|  |  |  |  | Baseline | Post-supplement |  |
| Romano *et al*, 1991 (Italy) | RCT | n= 35 from 1^st^ trimester | 120-128 µg/day (iodized salt) | 37 µg/L | 100 µg/L | Efficacy  *Maternal:*  *-*Urine Iodine Concentration ↑  -thyroid size ↓ (3/5 trials)  -Thyrotropin ↓ (2/6 trials)  -thyroid hormones =  *Neonatal:*  -thyroid growth ↓  - Thyroglobulin release ↓  -thyroid hormones =  -better neurodevelopmental outcomes, but effect may be lost after weeks 6-10.  Safety  *Maternal:*  -generally safe  -thyroid autoimmunity =  -Post-partum thyroid dysfunction prevalence/severity = (small sample)  -No data on long-term outcomes (goitre, autoimmunity)  *Neonatal:*  -generally safe  - Thyrotropin ↑ (2/6 trials)  - No data on birth weight, prematurity and long-term outcomes (thyroid autoimmunity, child development) |
| Pendersen*et al*, 1993 (Denmark) | RCT | n=54 from week 17 | 200 µg/day (KI solution) | 55 µg/L | 90-110 µg/L |  |
| Glinoer*et al*, 1995 (Belgium) | RCT | N=120 from week 14 | 100 µg/day (tablet) | 36 µg/L | 80-90 µg/L |  |
| Liesenkötter*et al*,1996 (Germany) | Quasi-RCT | N=108 from week 11 | 230 µg/day (tablet) | 53 µg/L | 104 µg/g Cr |  |
| Nøhr*et al*, 2000 (Denmark) | RCT | N=66 from week 11 | 150 µg/day (multi-nutrient tablet) | 50 µg/L | 105 µg/L |  |
| Antonangeli*et al*, 2002 (Italy) | Open label RCT, randomi-zation not described | N=67 from week 10-16 | 50 µg/day  200 µg/day  (tablet) | 65 µg/g Cr (19 µg/L) 18-26 weeks  91 µg/g Cr (38.2 µg/L) 29-33 weeks | 128 µg/g Cr (65.4 µg/L)  230 µg/g Cr (140.5 µg/L) |  |
| Berbel*et al*, 2009 (Spain) | Interven-tional study | N=345 from weeks 4-6, 12-14 or full term | 200 µg/day (tablet) | 74.6 µg/L | 96.7 µg/L  120.5 µg/L |  |
| Velasco *et al*, 2009 (Spain) | Interven-tional study | N=194 from 1^st^ trimester (mostly week 10) | 300 µg/day (tablet) | 153.4 µg/L  87.6 µg/L | 263 µg/L |  |
| Zimmermann *et al*, (Thailand/ India) | Multi-centre RCT | N=800 from < week 14 | 200 µg/day (tablet) | N/A | N/A |  |
| Brucker-Davis *et al*  (France) | RCT | N=110 from week 8 | 150 µg/day (multi-nutrient tablet) | N/A | N/A |  |

Sources: References 1−10 in this Additional file.

**Table 2. Summary of international recommendations on maternal oral iodine supplementation**

| Organization | Year | Country | Target groups | Iodine dose |
| --- | --- | --- | --- | --- |
| Australian Thyroid Association | 2005 | Australia/New Zealand | - Pregnancy  - Lactation | -100-200 μg/day  -100-200 μg/day |
| Spanish Ministry of  Health (+ UNICEF Spain) | 2006 | Spain | - Pregnancy  - Lactation  -Women contemplating pregnancy | - ≥ 200 μg/day  - ≥ 200 μg/day  - ≥ 200 μg/day |
| American Thyroid Association (ATA) | 2006 | United States/Canada | - Pregnancy  - Lactation | - 150 μg/day  - 150 μg/day |
| World Health Organization (WHO)  United Nations Children's Fund (UNICEF)  International Council for the Control of Iodine Deficiency Disorders (ICCIDD) | 2007 | Countries/regions where:  - < 90% households use iodized salt  -median UIC < 100 µg/L in school-age children | - Pregnancy  - Lactation  - Women of reproductive age | - 250 μg/day  - 250 μg/day  - 150 μg/day  Note: alternatively, annual single dose of iodized oil 400 mg in harder-to-reach women |
| National Health and Medical Research Council of Australia (NHMRC) | 2010 | Australia | - Pregnancy  - Lactation  - Women contemplating pregnancy | - 150 μg/day  - 150 μg/day - 150 μg/day |
| Macedonian Ministry of Health | 2009 | Macedonia | Not available | Not available |
| New Zealand Ministry of Health | 2010 | New Zealand | - Pregnancy  - Lactation | - 150 μg/day  - 150 μg/day |

Sources: References 15−24 in this Additional file.

Table 3. Comparison among pharmaceutical forms and formulations used for iodine supplementation

| **Formulation** | **Advantages** | **Disadvantages** |
| --- | --- | --- |
| Oral tablet, iodine-only (generally KI) | 1. Proven efficacy and safety in mild-to-moderate settings 2. Minor and short-term adverse effects 3. Recommended as first option by most organizations. 4. Even though tablets are generally more expensive than other forms, the main cost are those of the system used to deliver the programme. Countries are likely to save up to 5% GDP by investing only 0.3% GDP in any micronutrient programme (World Bank) | 1. Additional data on optimal iodine doses and duration of treatment is warranted. 2. Requires effective and sustainable programme management |
| Oral tablet, KI combined with folic acid/vitamin B_12_ | 1. Same as above (1-4) 2. Improved compliance vs. iodine-only tablet, as 3 essential micronutrients are provided in one tablet | 1. Same as above (1,2) 2. Different timeframe for taking iodine and folic acid and therefore an iodine-only tablet should also be funded |
| IM iodized oil | 1. A single dose delivers 480 mg with effect lasting for at least 1-3 years 2. Over the past 60-70 years, extensively used with few side effects, even fewer than with iodized salt 3. Seems to avoid high early iodide levels in comparison with oral iodized oil 4. Improved compliance vs. abovementioned options | 1. Same as above (1) 2. RCTs on its efficacy and safety carried out only in moderate-to-severely iodine-deficient settings 3. Theoretically may cause foetal hypothyroidism; cases iodine-induced hyperthyroidism in people aged over 45 years and those with nodular goitre 4. Potentially high initial doses (mg) and impossibility of discontinuing treatment if adverse effects occurs 5. Need to adjust with future daily iodine supplementation programmes 6. Risk of infections 7. Painful |
| Oral iodized oil | 1. Same as above (2) 2. A single dose covers for 6 -12 months 3. Simpler, cheaper and safer than injection, since it requires less training, instruments and time 4. Can be made at cheaper cost than injection 5. Improved compliance vs. abovementioned options | 1. Same as above (1-5) |
| Oral saturated solution of potassium iodide or Lugol’s solution | 1. Minor and short-lived adverse effects 2. Single oral doses of potassium iodide monthly (30 mg) or biweekly (8 mg) may provide adequate iodine (but this evidence from children) 3. Cheap and widely available in rural dispensaries | 1. Despite the evidence from one Danish RCT on efficacy and safety in a mild-to-moderate country, additional data on optimal iodine doses and duration of treatment warranted. 2. Requires sustainable and effective programme management 3. Risk of accidental overdosing and compliance issues 4. Not pleasant to taste and frequently causes some gastro-intestinal discomfort 5. Thermo-and photosensitive |
| Oral multinutrient preparation | 1. Possibility of supplement mothers with other necessary micronutrients 2. Lack of selenium, iron, and vitamin A may exacerbate the effects of iodine deficiency 3. Improved compliance when patients need other supplements 4. Improved compliance than abovementioned options when there is deficiency of several micronutrients | 1. Same as above (1,2) 2. Iodine content and bioavailability varies widely among preparations 3. Some micronutrients may not be appropriate for non-deficient mothers 4. A potential risk of interactions between micronutrients affecting absorption and bioavailability 5. Controversial evidence on the optimal doses and benefits of some micronutrients |

Sources: References 11−42 in this Additional file.

**References**

1. Romano R, Jannini EA, Pepe M, Grimaldi A, Olivieri M, Spennati P, et al. The effects of iodoprophylaxis on thyroid size during pregnancy. Am J Obstet Gynecol 1991; 164: 482–5.
2. Pedersen KM, Laurberg P, Iversen E, Knudsen PR, Gregersen HE, Rasmussen OS, et al. Amelioration of some pregnancy-associated variations in thyroid function by iodine supplementation. J Clin Endocrinol Metab 1993; 77: 1078–83.
3. Glinoer D, De Nayer P, Delange F, Lemone M, Toppet V, Spehl M, et al. A randomized trial for the treatment of mild iodine deficiency during pregnancy: maternal and neonatal effects. J Clin Endocrinol Metab 1995; 80:258–69.
4. Liesenkötter KP, Göpel W, Bogner U, Stach B, Grüters A. Earliest prevention of endemic goiter by iodine supplementation during pregnancy. Eur J Endocrinol 1996; 134: 443–8.
5. Nøhr SB, Jorgensen A, Pedersen KM, Laurberg P. Postpartum thyroid dysfunction in pregnant thyroid peroxidase antibody-positive women living in an area with mild to moderate iodine deficiency: is iodine supplementation safe? J Clin Endocrinol Metab 2000; 85: 3191–8.
6. Antonangeli L, Maccherini D, Cavaliere R, Di Giulio C, Reinhardt B, Pinchera A, et al. Comparison of two different doses of iodide in the prevention of gestational goiter in marginal iodine deficiency: a longitudinal study. Eur J Endocrinol 2002; 147: 29–34.
7. Berbel P, Mestre JL, Santamaría A, Palazón I, Franco A, Graells M, et al. Delayed neurobehavioral development in children born to pregnant women with mild hypothyroxinemia during the first month of gestation: the importance of early iodine supplementation.Thyroid 2009; 1: 511–9.
8. Velasco I, Carreira M, Santiago P, Muela JA, García-Fuentes E, Sánchez-Muñoz B, et al. Effect of iodine prophylaxis during pregnancy on neurocognitive development of children during the first two years of life. J Clin Endocrinol Metab 2009; 94: 3234–41.
9. ClinicalTrials.gov. Maternal iodine supplementation and effects on thyroid function and child development (MITCH) [online]. 2010 Jul 13 [cited 2010 May 20]; Available from: URL: http://clinicaltrials.gov/ct2/show/NCT00791466
10. ClinicalTrials.gov. Iodine Supplementation During pregnancy and neuropsychological development [online]. 2010 Jan 13 [cited 2010 Sept 20]; Available from: URL: http://clinicaltrials.gov/ct2/show/study/NCT01049659?cond=%22Iodine+Deficiency%22&rank=2
11. Zimmermann M, Delange F. Iodine supplementation of pregnant women in Europe: a review and recommendations. Eur J Clin Nutr 2004; 58 (7): 979-84.
12. Zimmermann MB. Iodine deficiency. Endocr Rev 2009; 30: 376-408.
13. [Zimmermann MB](http://www.ncbi.nlm.nih.gov/pubmed?term="Zimmermann MB"%5BAuthor%5D). Iodine deficiency in pregnancy and the effects of maternal iodine supplementation on the offspring: a review. [Am J Clin Nutr.](javascript:AL_get(this, 'jour', 'Am J Clin Nutr.');) 2009; 89(2):668S-72S.
14. Puig-Domingo M, Vila L. Iodine status, thyroid and pregnancy. Hot Thyroidol [serial online] 2010 May [cited 2010 Jul 7]; 5/10. Available from: URL: http://www.hotthyroidology.com/editorial_pdf/HT_5_10.pdf
15. Casimiro-Soriguer FJ, Arena J, Orera M, Rodríguez MA, Bailón E, Gallo M. Guía para la prevención de defectos congénitos [Guide for the prevention of congenital defects]. Madrid: Ministry of Health and Consumers Affairs; 2006. Spanish
16. Vila L. Avances en la erradicación de la deficiencia de yodo en España [Progress in eradication of iodine deficiency in Spain] (editorial). Endocrinol Nutr 2010; 57 (3): 87–9. Spanish
17. Public Health Committee of the American Thyroid Association, Becker DV, Braverman LE, Delange F, Dunn JT, Franklyn JA, *et al*. Iodine supplementation for pregnancy and lactation-United States and Canada: recommendations of the American Thyroid Association. Thyroid. 2006;16 (10): 949-51.
18. WHO, UNICEF. Reaching optimal iodine nutrition in pregnant and lactating women and young children. Joint Statement of the World Health Organization and the United Nations Children's Fund. Geneva: World Health Organization; 2007.
19. WHO, UNICEF, ICCIDD. Assessment of iodine deficiency disorders and monitoring their elimination: a guide for programme managers. 3rd ed. Geneva: World Health Organization; 2007.
20. Pinchera A. Progress against IDD in Europe. IDD newsletter [serial online] 2010 May [cited 2010 August 31]; 35 (2): 4-8. Available from: URL: http://www.iccidd.org/media/IDD%20Newsletter/2007-present/IDD_newsletter-may10.pdf
21. National Health and Medical Research Council. Iodine Supplementation for Pregnant and Breastfeeding Women. NHMRC public statement. January 2010 [online]. Available from: URL: http://www.nhmrc.gov.au/_files_nhmrc/file/publications/synopses/new45_statement.pdf Canberra: NHMRC; 2010 [cited 2010 Jul 10].
22. Ministry of Health of New Zealand. Maternity: supplement (tablet) to take when pregnant or breastfeeding. [online] Available from:URL: http://www.moh.govt.nz/moh.nsf/indexmh/pregnancy-supplement Wellington: Ministry of Health; 2010 [cited 2010 Jul 10]
23. Morreale de Escobar G , Escobar del Rey F. Five years of progress against IDD in Spain. IDD newsletter [serial online] 2008 Aug [cited 2010 Jul 7]; 29 (3): 10-11. Available from: URL: http://www.iccidd.org/media/IDD%20Newsletter/2007-present/IDD%20NL%20aug08.pdf
24. Ministry of Health and Pharmaceutical Management Agency of New Zealand. Newly funded iodine only tablet to meet unmet need. Media release. 2010 Jul 1[online]. Available from: URL: http://www.moh.govt.nz/moh.nsf/0/C51381CC145A60F8CC2577520077CA4D Wellington: Ministry of Health; 2010 [cited 2010 Jul 7].
25. Andersson M, de Benoist B, Darnton-Hill I, Delange F. Iodine deficiency in Europe: a continuing public health problem. Geneva: World Health Organization; 2007.
26. Goodall S, Norman R, Gallego G. Cost-effectiveness analysis of alternate strategies to redress iodine deficiency in Australia. [online] Available from: URL: http://www.foodstandards.gov.au/_srcfiles/P1003%20SD2%20-DoHa%20CHERE%20report%20-%20cost-effectiveness%20analysis.pdf Sydney: Centre for Health Economics Research and Evaluation; 2007 [cited 2010 Jul 10].
27. Delange F. Administration of iodized oil during pregnancy: a summary of the published evidence. Bull World Health Organ 1996; 74 (1): 101–8.
28. Untoro J, Timmer A, Schultink W. The challenges of iodine supplementation: a public health programme perspective. Best Pract Res Clin Endocrinol Metab. 2010;24 (1) :89-99.
29. Lumley J, Watson L, Watson M, Bower C. Periconceptional supplementation with folate and/or multivitamins for preventing neural tube defects. (Cochrane Review) In: Cochrane Library, Issue 3, Oxford: Update Software; 2001.
30. Haider BA, Bhutta ZA. Multiple-micronutrient supplementation for women during pregnancy. (Cochrane Review) In: Cochrane Library, Issue 4, Oxford: Update Software; 2006.
31. Benmiloud M, Delange F, Pittman CS,Yaffe S, Thilly C, Voumard C, *et al*. Safe use of iodized oil to prevent iodine deficiency in pregnant women. A statement by the World Health Organization. Bull World Health Organ. 1996; 74 (1): 1-3.
32. Kochupillai N. Prevention and control of goitre. Demerits of iodised oil injections. NFI Bull. 1991; 12: 1-5.
33. Wolff J. Physiology and pharmacology of iodized oil in goiter prophylaxis. Medicine (Baltimore). 2001; 80 (1): 20-36.
34. Hetzel BS, Delange F, Stanbury JB, Viteri FE. The Prevention and Control of Iodine Deficiency Disorders – Nutrition policy discussion paper No. 3. New York: United Nations; 1988.
35. Frazier CH. The use of potassium iodide in hyperthyroidism. Ann Surg. 1932; 95 (4): 517–24.
36. Upsher-Smith. SSKI Patient Information leaflet. Minneapolis, MN: Upsher-Smith Laboratories.
37. WHO/SEARO. Elimination of iodine deficiency disorders in South-East Asia. Report of a regional consultation. New Delhi, 24-26 February 1997. New Delhi, India: World Health Organization Regional Office for South East Asia; 1997.
38. Hess SY. The impact of common micronutrient deficiencies on iodine and thyroid metabolism: the evidence from human studies. Best Pract Res Clin Endocrinol Metab. 2010; 24 (1): 117-32.
39. Sandström B. Micronutrient interactions: effects on absorption and bioavailability. Br J Nutr. 2001; 85 (Suppl 2): S181-5.
40. Azaïs-Braesco V, Pascal G. Vitamin A in pregnancy: requirements and safety limits. Am J Clin Nutr. 2000; 71(5 Suppl): 1325S-33S.
41. Eneroth H, El Arifeen S, Persson LA, Lönnerdal B, Hossain MB, Stephensen CB, *et al*. Maternal multiple micronutrient supplementation has limited impact on micronutrient status of Bangladeshi infants compared with standard iron and folic acid supplementation. J Nutr. 2010; 140 (3): 618-24.
42. Roberfroid D, Huybregts L, Lanou H, Henry MC, Meda N, Kolsteren F P; Micronutriments et Santé de la Mère et de l'Enfant Study (MISAME) Group. Effect of maternal multiple micronutrient supplements on cord blood hormones: a randomized controlled trial. Am J Clin Nutr. 2010; 91 (6): 1649-58.
