# Supplementary material for: Lessons drawn from research utilization in the maternal iodine supplementation policy development in Thailand
Source: BMC Public Health. 2012 May 30;12:391. doi: 10.1186/1471-2458-12-391 (PMC3490728; doi:10.1186/1471-2458-12-391)
Supplement: Additional file 2 — Summary of the rapid review results [file 1471-2458-12-391-S2.doc]

**Additional file 2. Summary of the rapid survey results**


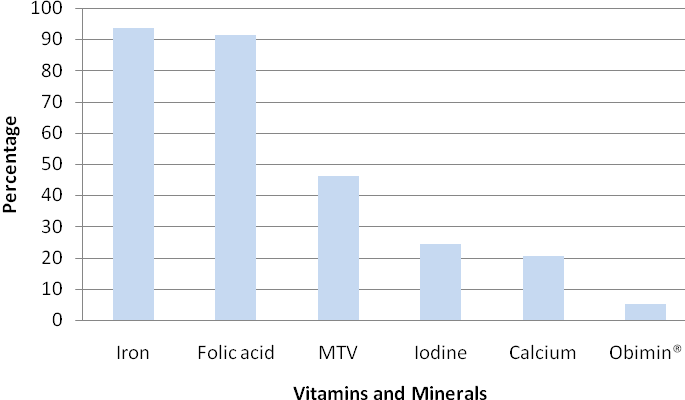
**Figure 1. Current vitamin and mineral supplements provided by obstetricians**

MTV: multinutrient (“multivitamin”) preparation

Note:Obimin® may not be available in other countries

**Figure 2. Awareness of vitamin and mineral deficiency**


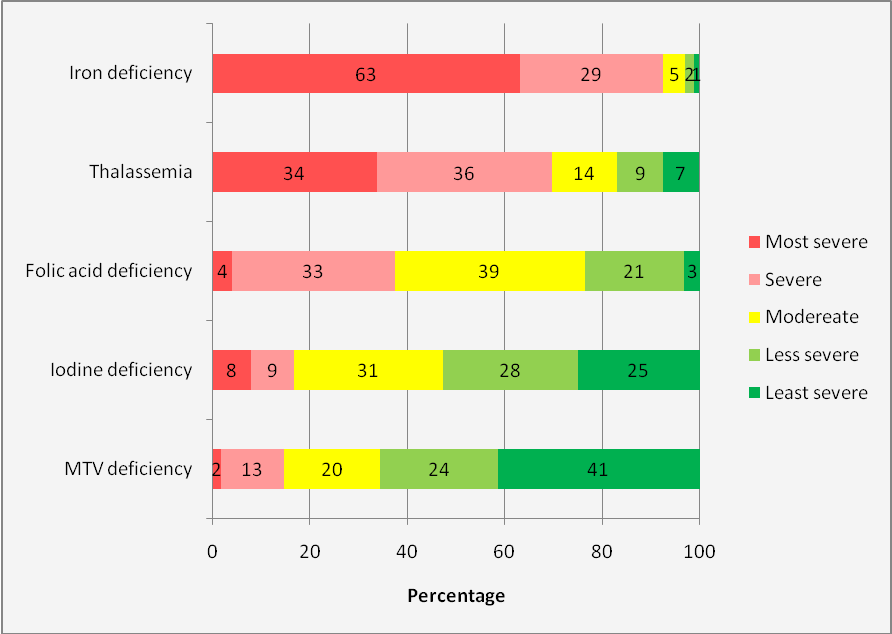


MTV: Multinutrient (“multivitamin”).

Table 1. Preferable formulation of iodine tablet supplement among Thai obstetricians

| **Formulations** | **Percentage** | **Reasons** |
| --- | --- | --- |
| 1. Potassium iodide (Iodine GPO®) | 3 | Easy to adjust dosage |
| 1. Potassium iodide + folic acid | 4 | Suitable for thalassemic cases, no iron that can stimulate nausea and vomiting |
| 1. Potassium iodide + folic acid + iron (Triferdine®) | 80 | Optimal compliance, all necessary nutrients are included, optimal drug administration, can solve vitamin and mineral deficiencies among pregnancies, iron is an essential supplement for pregnant women |
| 1. None of above | 13 | Need more information, such as prevalence of iodine deficiency and drug indications, fear of drug interactions with the current multinutrient supplements, no perception of iodine deficiency |

**References**

Tonmukayakul U, Vivekmetakorn S, Teerawattananon Y. [A survey of maternal vitamin and mineral supplements prescribed by Thai obstetricians]. Journal of Health Systems Research. 2011; 5(1): 40-6. Thai
